# Long-read sequencing enables high-accuracy mitochondrial heteroplasmy detection in Parkinson’s disease

**DOI:** 10.1186/s12864-026-13426-y

**Published:** 2026-10-08

**Authors:** Theresa Lüth, Susen Schaake, Christoph Much, Mary Megan Belyea, Philip Seibler, Anne Grünewald, Patrick May, Christine Klein, Hansi Weissensteiner, Joanne Trinh

**Affiliations:** 1 Institute of Neurogenetics, University of Lübeck, Lübeck, Germany; 2 Luxembourg Centre for Systems Biomedicine, University of Luxembourg, Esch-sur-Alzette, Luxembourg; 3 Institute of Genetic Epidemiology, Medical University of Innsbruck, Innsbruck, Austria

**Keywords:** PacBio sequencing, Long-read, mtDNA, Heteroplasmy, Benchmarking, Mixtures, Low-frequency variant, Parkinson''s disease, LRRK2

## Abstract

**Background:**

Low-frequency heteroplasmic mitochondrial DNA (mtDNA) variants are associated with aging and neurological diseases, including Parkinson’s disease (PD). Targeted deep mtDNA sequencing using PacBio HiFi long reads has the potential to resolve heteroplasmy across the full mitochondrial genome with high accuracy.

**Methods:**

To validate Vega PacBio sequencing for detecting mtDNA heteroplasmy, we analyzed four predefined mixtures of two mtDNA haplotypes. We generated a single long-range PCR amplicon covering the entire mitochondrial genome. These amplicons were mixed at predefined ratios (minor mixture haplotype component: 5%, 2%, 1%, and 0.1%). Variant calling was performed using *Mutserve2*, and accuracy was assessed by calculating the F_1_ score from comparisons between expected and detected variants. Full-length mtDNA PacBio sequencing was applied to explore heteroplasmy across fibroblast passages derived from five LRRK2 p.Gly2019Ser variant carriers (*n* = 3 affected with PD and *n* = 2 unaffected carriers). Changes in mtDNA heteroplasmy level and variant load were assessed longitudinally using a linear mixed model as a proof-of-principle.

**Results:**

The single-amplicon approach enabled full-length haplotype resolution without amplification bias associated with overlapping PCR strategies. The F_1_ score of the predefined mixtures was 1.0 for heteroplasmy levels between 5% and 1% and remained high (0.91) at 0.1%. At the specifically lowered calling threshold applied to the 0.1% mixture, *n* = 10/62 additional variants discordant with the Illumina reference were observed, but sensitivity remained very high at 1.00 in that mixture. Detected minor variants closely matched expected heteroplasmy levels, with average variant levels of 0.057 (5%), 0.022 (2%), 0.011 (1%), and 0.001 (0.1%). Across twelve fibroblast passages, we observed fewer mtDNA heteroplasmic variants. In this small proof-of-principle experiment, heteroplasmic variant load was higher in affected (*n* = 3) than in unaffected (*n* = 2) *LRRK2* variant carriers and with older age. Notably, we observed distinct patterns of heteroplasmic variants that either increased or decreased in heteroplasmy level across passages.

**Conclusion:**

PacBio HiFi sequencing, combined with a single-amplicon strategy, enables accurate full-length mtDNA heteroplasmy detection and longitudinal analysis, providing a valuable tool for studying mitochondrial variation and dynamics in disease.

**Supplementary information:**

The online version contains supplementary material available at https://doi.org/10.1186/s12864-026-13426-y.

## Background

Ongoing mutation and selection of mitochondrial DNA (mtDNA) in both the germline and soma generate mtDNA heteroplasmy - the coexistence of multiple different mitochondrial genotypes within a cell, tissue, or organism. Damage repair in mtDNA is less efficient than in nuclear DNA, resulting in an ~ 10× higher mutation rate. Consequently, not all mtDNA copies share the same variants; those present in only a subset are called heteroplasmic variants [1, 2]. It has been reported that mtDNA heteroplasmy is not only closely linked to aging [3], but also implicated in a range of diseases, including neurodegenerative disorders such as Parkinson’s disease (PD) [4–6]. Therefore, accurate characterization and quantification of mtDNA heteroplasmic variants are essential for understanding mitochondrial biology and the role of mtDNA heteroplasmy in disease.

Next-generation, short-read sequencing technologies have enabled high-depth mtDNA analysis [3] and are currently considered the gold standard for heteroplasmy detection [7]. However, these approaches rely on read lengths of ~ 50-300bps, which can lead to uneven coverage, mapping biases, vulnerability towards NUMTs contamination, and limit the direct resolution of full-length mtDNA haplotypes. Additionally, longer read lengths can facilitate the assessment of structural variants, as reads can span larger genetic events.

Recent studies have demonstrated the utility of long-read sequencing for mtDNA analysis, including the characterization of structural variants [8], de novo mitochondrial genome assembly, haplotype reconstruction [9], and targeted multi-amplicon mtDNA sequencing [9, 10]. These studies highlight the advantages of long-read technologies for resolving the mitochondrial genome but have generally focused on genome assembly, deletion detection, or higher-level heteroplasmies. Thus, it has been demonstrated that long-read sequencing technologies have the potential to overcome several limitations of short-read sequencing. It has been demonstrated that Oxford Nanopore long-read sequencing can accurately detect mtDNA heteroplasmy [11, 12]. In our previous benchmarking study using Oxford Nanopore sequencing (R9.4.1 chemistry and Guppy v5.0.11 basecalling), accurate mtDNA heteroplasmy detection was achieved for variant levels above 5% [11, 12]. Although ONT sequencing chemistry and basecalling models have substantially improved since then, systematic benchmarking of these advances for low-frequency mtDNA heteroplasmy detection remains limited. PacBio HiFi sequencing provides highly accurate long reads, making it an interesting tool for detecting low-level variants. Still, systematic benchmarking of PacBio long-read sequencing for accurate mtDNA heteroplasmy detection remains limited, particularly at low variant levels (< 5%). Additionally, the deep sequencing of full-length mtDNA molecules has often been compromised by the use of multiple overlapping PCR amplicons [5, 7, 11].

In this study, we explored the performance of PacBio HiFi sequencing for detecting mtDNA heteroplasmy using a single full-length long-range PCR approach. Utilizing predefined haplotype mixtures, we assessed detection accuracy across low variant levels. Finally, we applied this approach to investigate longitudinal heteroplasmic dynamics in fibroblast passages from LRRK2 p.Gly2019Ser variant carriers, affected by PD or unaffected.

## Methods

### Study design and sample overview

**Fig. 1:**
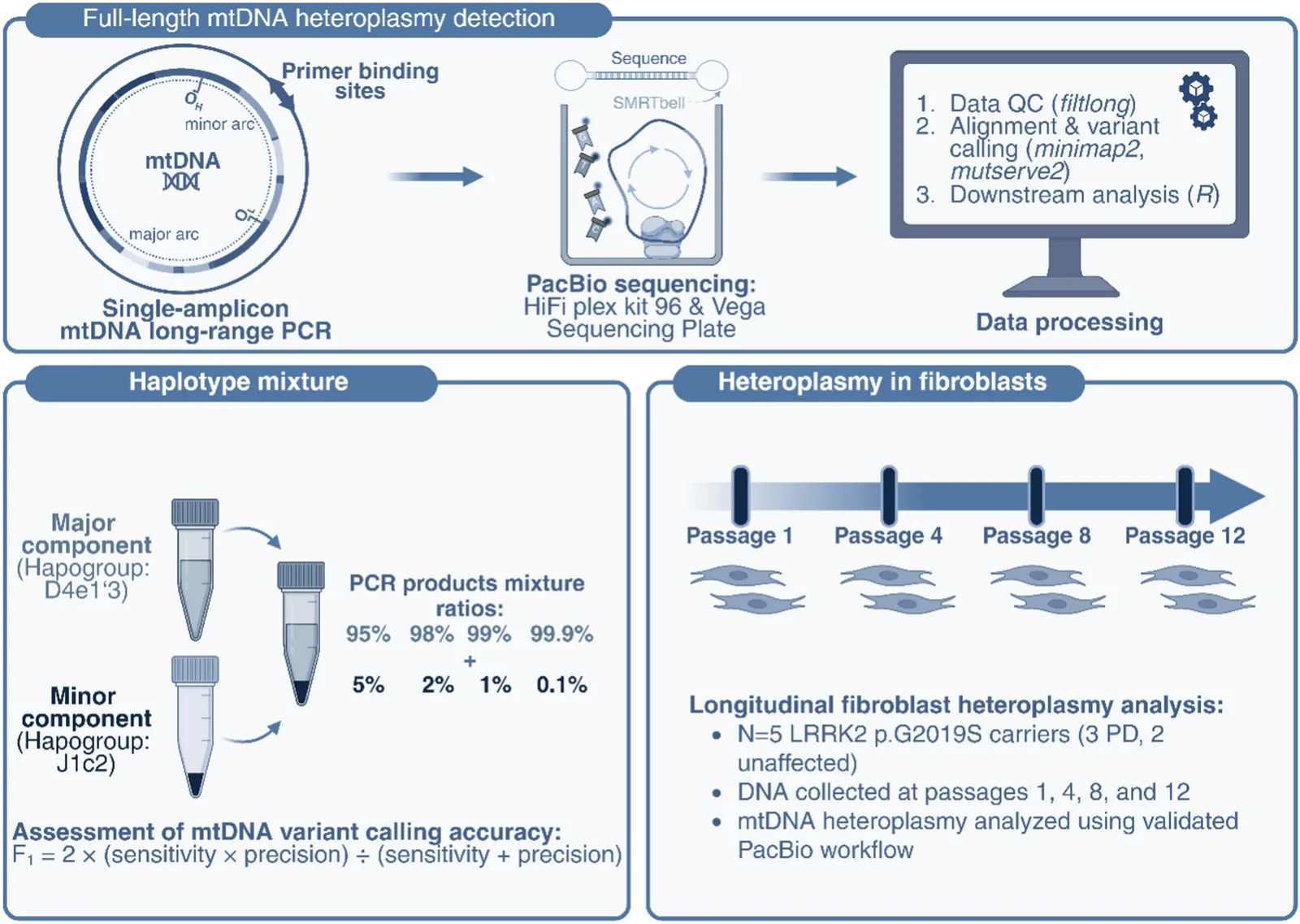
Study overview and workflow for full-length mtDNA heteroplasmy detection using PacBio HiFi sequencing**.** A single long-range PCR amplicon spanning the entire mitochondrial genome was generated and sequenced using the PacBio HiFi platform. Sequencing reads were subjected to quality control, alignment, and heteroplasmy detection using *minimap2* and *Mutserve2*, followed by downstream statistical analyses in R. To validate heteroplasmy detection accuracy, predefined mixtures of two mtDNA haplotypes were generated at ratios ranging from 5% to 0.1% and evaluated using the F1 score. The validated workflow was subsequently applied to investigate longitudinal mtDNA heteroplasmy dynamics across fibroblast passages (1, 4, 8, and 12) derived from five LRRK2 p.G2019S variant carriers. Created in BioRender. Lüth, T. (2026) https://BioRender.com/jzuzocu

To evaluate the applicability of PacBio HiFi sequencing for detecting mtDNA heteroplasmy across the full mitochondrial genome, we used a two-part study design consisting of a mixture-based validation experiment and a biological application (Fig. 1).

First, predefined mixtures of two mtDNA haplotypes were generated to benchmark heteroplasmy detection accuracy across a range of low-frequency variant levels. As previously described, the DNA used in the mixture model was extracted from blood with the Blood and Cell Culture DNA Midi kit (Qiagen), and the selected samples were from participants of European ancestry [11] (Supplementary Table 1). These mixtures were prepared at defined proportions ranging from 95% (major component, haplogroup D4e1’3) plus 5% (minor component, haplogroup J1c2) to 99.9% (major component) plus 0.1% (minor component). The samples were selected based on their haplotypes, containing both shared variants and variants that differ between the two haplogroups. The samples had previously been characterized using Illumina NextSeq sequencing as a gold-standard method, allowing us to assess the accuracy of our workflow by comparing expected and observed variants, particularly those from the minor component that mimic mitochondrial heteroplasmy [11].

Second, the validated sequencing workflow was applied to investigate longitudinal heteroplasmic changes across time as proof-of-principle. We used fibroblast cell lines derived from five LRRK2 p.Gly2019Ser variant carriers (*n* = 3 affected with PD and *n* = 2 unaffected carriers, Supplementary Table 1). The rationale for selecting *LRRK2*-related cellular models is based on previous reports on mitochondrial mechanisms in LRRK2 penetrance [13–15]. For this purpose, mtDNA was extracted from fibroblast cultures at passages 1, 4, 8, and 12 to assess changes in heteroplasmic variant load and heteroplasmy levels across cell passages longitudinally. In both experimental settings, deep mtDNA PacBio sequencing was performed using a single long-range PCR amplicon spanning the entire mitochondrial genome.

### Long-range PCR amplification of mtDNA

One single amplicon spanning the complete mtDNA was prepared with long-range PCRs. The primer set binds within the minor arc of the mtDNA (chrM:2120 F: 5′- GGA CAC TAG GAA AAA ACC TTG TAG AGA GAG − 3′ and chrM:2119R: 5′- AAA GAG CTG TTC CTC TTT GGA CTA ACA − 3′) [16]. We used the high-fidelity Platinum SuperFi II DNA Polymerase, and the details for the PCR mix and conditions are listed in Supplementary Table 2.

### Preparation of haplotype mixture

The DNA concentration of the resulting PCR products was measured using a Qubit fluorometer and subsequently normalized. To generate the haplotype mixtures, PCR products of the major component (sample B-28, haplogroup D4e1′3) were combined with PCR products of the minor component (sample L-2804, haplogroup J1c2) at predefined proportions with minor mixture components of 5%, 2%, 1%, and 0.1% (Supplementary Table 3).

### LRRK2 variant carrier-derived fibroblast cultures

We included five fibroblast cell lines derived from carriers of the LRRK2 p.Gly2019Ser variant. All cell lines had previously been expanded and cryopreserved at passage 3. For the present study, all fibroblast lines were thawed simultaneously and cultured in parallel in T75 flasks using DMEM supplemented with 1% Penicillin-Streptomycin and 10% FBS. After reaching confluency, cells were passaged every seven days. Samples for DNA extraction were collected after the first passage following thawing (defined here as passage 1) and subsequently after passages 4, 8, and 12. At each time point, half of the cells were harvested for DNA extraction, while the remaining cells were continued in culture until the next sampling time point. The aliquot was centrifuged at 1000xg for 5 min at 4 °C, and the supernatant was removed. The pellet was then washed once in DPBS, and after another centrifugation, the DPBS was removed, and the pellets were stored at -80 °C until DNA extraction. The DNA was extracted using the AutoGen XTRACT 16 + XK401 Kit for cultured cells, and the DNA quality was measured with the Qubit fluorometer and the Nanodrop. Subsequently, the mtDNA long-range PCR was performed for all fibroblast-derived DNA samples as described above.

### PacBio library preparation and sequencing

All four mixture samples and all PCR products (DNA) derived from the fibroblasts were barcoded and sequenced together. We used 480ng of PCR-amplified DNA as the input for the library preparation, starting with the end repair, following the manufacturer’s instructions of the HiFi plex kit 96 (103-381-300) with the SMRTbell adapter index plate 96 A (102-009-200) and the Vega polymerase kit (103-426-500). The sequencing was performed on the PacBio Vega device with the Vega Sequencing Plate (103-274-300) and the Vega SMRT cell tray (103-406-700). The sequencing run comprised a total of 40 barcoded samples on a single PacBio Vega SMRT Cell, including the 24 samples analyzed in this study and 16 additional mtDNA sequencing samples that were unrelated to the present work.

### Sequencing data processing

PacBio sequencing data generated on the Vega system were obtained as unmapped BAM files. Approximately 95% of the sequenced reads were classified as HiFi reads (base quality≥Q30). Reads were filtered using *Filtlong* (v0.2.1) (https://github.com/rrwick/Filtlong) to retain sequences with lengths between 15 kb and 18 kb. Filtered reads were aligned to the mitochondrial reference genome (revised Cambridge Reference Sequence, rCRS) using *Minimap2* (v2.22) [17]. The resulting alignments were subsequently sorted and indexed using *Samtools* (v1.3.1) [18] and filtered for alignment lengths >1 kb.

Our group previously benchmarked multiple alignment and variant-calling tools for assessing mtDNA heteroplasmy using Oxford Nanopore long-read sequencing, demonstrating that *Mutserve2* provided the highest variant-calling accuracy, while alignment tools had only a marginal impact on performance [11]. Therefore, we used *Mutserve2* (v2.0.0-rc8) [19, 20], a tool specifically designed to detect heteroplasmic and homoplasmic mtDNA variants, for variant calling. Detected variants were annotated using the same tool, and potential sample contamination was assessed using *Haplocheck* (v1.3.3) [21]. To evaluate heteroplasmy detection accuracy across different sequencing depths in haplotype mixtures, we generated stepwise-downsampled subsets of the alignments using the *Samtools -s* option. In addition, we utilized *Himito* [22] a graph-based software tool for mtDNA analysis, to explore possible NUMTs contamination in the long-read data.

For the longitudinal fibroblast analysis, variant calling was performed using the complete sequencing dataset and the same data analysis workflow. Additionally, we used *Mitorsaw* (https://github.com/PacificBiosciences/mitorsaw) and *Sniffles2* [23] to call variants beyond SNVs in the fibroblasts. *Mitorsaw* is a variant caller specifically designed to call mtDNA variants from PacBio data, creating consensus sequences for each haplotype and performing an alignment to the looped version of the reference mitochondrial genome, and we used the *--minimum-maf 0.01* parameter. *Sniffles2* is a variant caller for long-read sequencing data, and we used the *--mosaic* and *--mosaic-af-min 0.01* parameters.

### Statistical analysis

As our group previously described [8], to evaluate variant-calling performance, detected variants were classified as true positives, false positives or false negatives. True-positive variants were defined as those expected to be present in the predefined haplotype mixtures based on the previously generated Illumina NextSeq sequencing data used as the reference standard. False-positive variants were defined as variants detected by the PacBio workflow but discordant from the Illumina reference dataset, whereas false-negative variants were variants expected in the mixtures but not detected. Based on these classifications, the F_1_ score was calculated as a combined measure of precision and sensitivity (F_1_ score = 2 × (precision × sensitivity) ÷ (precision + sensitivity)). To benchmark workflow performance across different biologically relevant heteroplasmy levels, mixture-specific minimum heteroplasmy thresholds were applied according to the expected minor-component fraction. This design reflects the intended analytical use of the workflow, where variant-calling thresholds are selected based on the heteroplasmy range of interest. The following minimum heteroplasmy thresholds were applied to the *Mutserve2* output: ≥0.025 for the 5% mixture, ≥ 0.010 for the 2% mixture, ≥ 0.005 for the 1% mixture, and ≥ 0.0005 for the 0.1% mixture. Consequently, performance across mixture levels was evaluated using mixture-specific thresholds and should not be interpreted as a direct comparison of analytical detection limits. In addition, the number of false-positive variants was calculated to assess further variant-calling performance. Variants within the primer-binding regions were excluded from interpretation. For the mixtures, we can differentiate between three different types of variants: (1) major variants (*n* = 20; only present in the major component and expected at 95%-99.9%), (2) minor variants (*n* = 18; only present in the minor component and expected at 5%-0.1%), and (3) common variants (*n* = 14; present in both samples and expected at 100% at all mixture ratios). All three variant classes were included in the calculation of sensitivity, precision, and F_1_ score, while the number of detected minor variants was additionally reported separately to assess recovery of the minor component.

Lastly, changes in mtDNA variant load across fibroblast passages were assessed using a linear mixed-effects model. Passage number, age at extraction, and PD status were included as fixed effects, while the fibroblast cell line was modeled as a random effect. The analyses were performed in *R* (v.4.3.1) [24] with the *lme4* (v.1.1–34) [25] and *lmerTest* (v.3.1-3) [26] packages. The mixed model was exploratory, and the resulting p-values are reported descriptively and should not be interpreted for significance.

## Results

First, we evaluated the sequencing performance across all multiplexed samples sequenced for this study. PacBio HiFi sequencing generated high-quality full-length mtDNA reads across all haplotype mixtures and fibroblast samples. More than 95% of all sequenced DNA reads achieved a basecalling quality Phred score of at least 30, corresponding to an accuracy of 99.9%. Filtering for q-score, read and alignment length, ~ 21% of the sequenced reads were discarded, mostly due to the read length (Supplementary Fig. 1). An exemplary gel image of the long-range PCR products showed a single band of the expected size without detectable nonspecific amplification. After applying the described filtering steps, the majority of the reads showed a read length of ~ 16.6 kb, representing the full-length mtDNA amplicon (Supplementary Table 4,Supplementary Fig. 1).

High sequencing depth was obtained across all 24 samples, with an average coverage of 83,180X (range: 64,035X-107,279X) after filtering for read and alignment lengths. Coverage was highly uniform across all positions of the mitochondrial genome, indicating even amplification and sequencing of the full-length amplicon (Supplementary Fig. 2A, exemplary sample). We randomly subsampled 0.5% of the sequenced reads and observed similarly even coverage across the entire mitochondrial genome at an approximate sequencing depth of 320X (Supplementary Fig. 2B).

### PacBio workflow is highly accurate for mtDNA variant detection in mixture models

To evaluate the accuracy of mtDNA heteroplasmy detection using the PacBio workflow, we analyzed predefined mixtures of two mtDNA haplotypes over a range of minor-haplotype fractions. Variant calling was performed using *Mutserve2* and compared with the expected variant profiles derived from the Illumina NextSeq reference data, as a gold-standard method. Across mixture levels from 5% to 1%, heteroplasmic variants were detected with perfect concordance between expected and observed variants, resulting in an F_1_ score of 1.0 (Table 1). Thus, we also detected no additional variants discordant with the Illumina reference in the 5%, 2%, or 1% mixtures.

**Table 1:** Summary of the coverage and accuracy measures detected from the mixture models

Minor component mixture fraction	Mean sequencing depth (SD)	Detected minor variants/expected minor variants	Additional variant calls	F_1_ scores
**5%**	64035X (543)	18/18	0	1.00
**2%**	66009X (560)	18/18	0	1.00
**1%**	75178X (641)	18/18	0	1.00
**0.1%**	70154X (596)	18/18	10	0.91

F_1_ score = 2 × (precision × sensitivity) ÷ (precision + sensitivity); Sensitivity = (Number of true positive calls) ÷ (Number of true positive calls + Number of false negative calls); Precision = (Number of true positive calls) ÷ (Number of true positive calls + Number of false positive calls)

To explore the recovery of variants at very low heteroplasmy levels, we additionally included a mixture containing 0.1% of the minor component, applying a lower minimum heteroplasmy threshold (i.e., 0.0005 for the 0.1% mixture). Under these conditions, we recovered all expected minor-haplotype variants; however, 10 additional variants passed the applied calling threshold, resulting in an F_1_ score of 0.91.

**Fig. 2:**
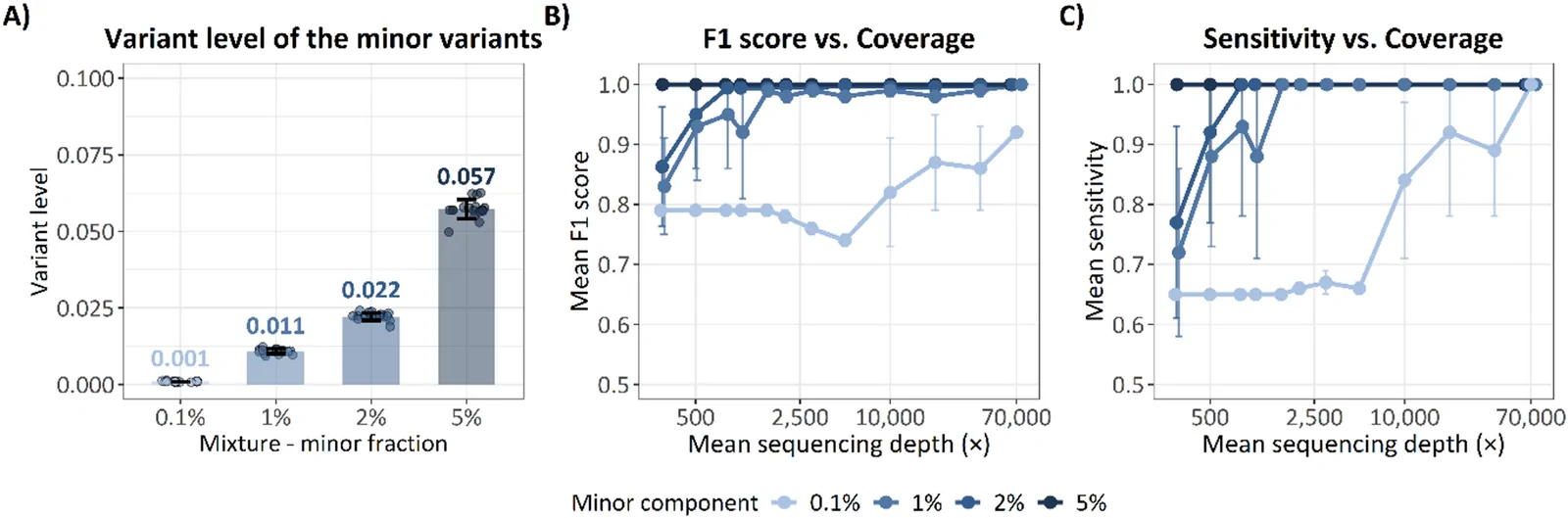
Validation of mtDNA heteroplasmy detection accuracy using predefined haplotype mixtures. (**A)** Comparison of the expected and detected heteroplasmy levels of informative minor mtDNA variants across the 5%, 2%, 1%, and 0.1% haplotype mixtures. Each dot represents an individual heteroplasmic variant detected by *Mutserve2*, while bars indicate the mean ± standard deviation. (**B)** F1 score and (**C**) sensitivity of heteroplasmy detection across a range of mean sequencing depths generated by systematic downsampling of the PacBio HiFi sequencing data. For each target sequencing depth, five independent random subsampling replicates were generated, and mean performance metrics ± standard deviation are shown. Performance was evaluated for haplotype mixtures containing minor component fractions of 5%, 2%, 1%, and 0.1%.

Importantly, the detected variant levels of minor variants across all mixtures closely matched the expected levels based on the predefined mixture ratios, with minimal deviation among individual variants (Fig. 2A).

Given the high sequencing depth obtained across all multiplexed samples, we next evaluated variant-calling accuracy and sensitivity at different decreasing sequencing depths by stepwise subsampling the data (Fig. 2B-C). We subsampled the sequencing data five times at each target coverage (i.e., eleven target coverages from 40,000X to 300X). Accuracy and sensitivity remained very high at sequencing depths > 1500X (F_1_ score range: 0.98-1.00, sensitivity = 1.00, Supplementary Table 5) for mixture ratios ≥ 1%. The F_1_ score and sensitivity remained 1.00 across all tested sequencing depths for the 5% mixture, even at the lowest evaluated coverage of approximately 300X.

Lastly, we utilized *Himito* to explore the indication of NUMTs contamination in all four mixtures. Using default parameters of the toolkit, there was no evidence for any NUMTs contamination in the data set.

### No detectable systematic sequencing errors and minimal sequencing noise

Type I errors (i.e., variants discordant with the Illumina reference) were investigated following the removal of all expected variants and were observed exclusively in the 0.1% haplotype mixture. Further characterization using the reference genome GRCh38 showed that these variants were not due to systematic errors on specific sites and were partially detected in low-complexity regions, such as short homopolymers (Supplementary Table 6). Additionally, we used *Mitorsaw*, a haplotype-aware variant caller, to assess the context of these additional variant calls in the 0.1% mixture. Interestingly, only two out of the ten additional variants discordant from the Illumina reference were also detected with *Mitorsaw* at a similar variant level as *Mutserve2*, and they did not cluster on the same haplotype (i.e., chrM:299 A and chrM:1419 A).

Common variants shared between major and minor components are expected to be consistently detected at a homoplasmic level of 1. As such, they serve as internal controls for assessing technical noise within the dataset, where any deviation from 1 would reflect technical or stochastic variation. These variants were further evaluated within the 5% haplotype mixture to validate the accuracy of the sequencing workflow (Supplementary Table 7). Across the full data set (~ 64,000 sequencing depth), variant levels remained at 1, and a high level of concordance was maintained after subsampling to approximately 300X depth, with no meaningful deviation observed (variant level ≥ 0.997).

### Read-level haplotype assignment validates full-length mtDNA phasing

To demonstrate the ability of PacBio HiFi sequencing to resolve complete mitochondrial haplotypes, individual full-length reads were classified according to informative major- and minor-haplotype markers (i.e., their discordant variants). Across all predefined haplotype mixtures (5%, 2%, 1%, and 0.1% minor component), the observed proportions of minor-haplotype reads closely matched the expected mixture ratios and reported variant levels from Mutserve2 (Supplementary Table 8, Fig. 2A). Visualization of representative reads from the 1% mixture demonstrated that informative variants consistently co-occurred on individual sequencing molecules, confirming reconstruction of full-length mitochondrial haplotypes (Supplementary Fig. 3).

### Proof-of-principle: mitochondrial heteroplasmy assessment in fibroblasts derived from LRRK2 variant carriers

**Fig. 3:**
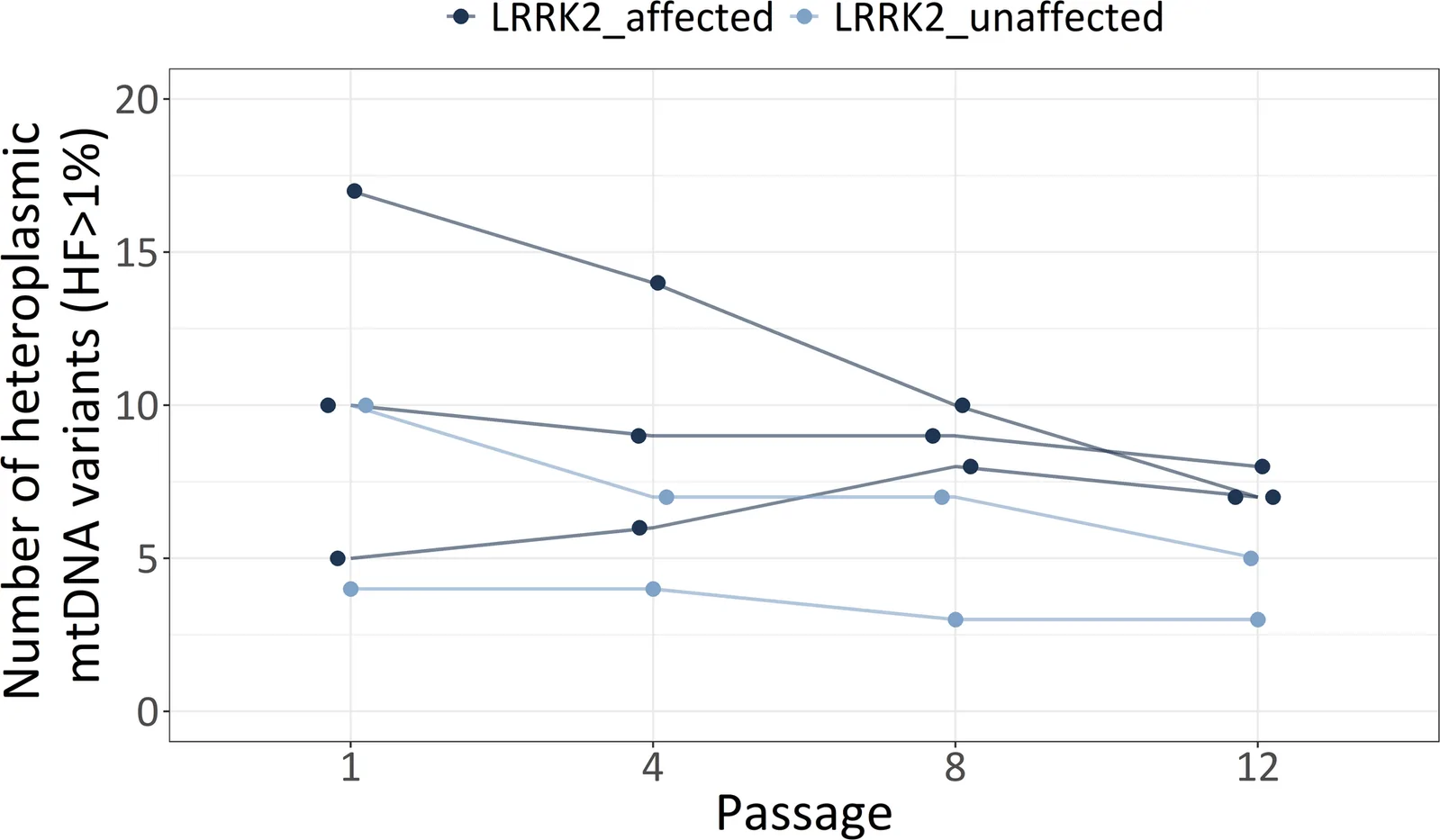
Longitudinal changes in heteroplasmic variant load across fibroblast passages in LRRK2 variant carriers. The number of mtDNA variants with heteroplasmy levels > 1% was quantified across fibroblast passages (1 (day 0), 4 (day 28), 8 (day 56), and 12 (day 84)) derived from five LRRK2 p.G2019S variant carriers, including three individuals affected with Parkinson’s disease (dark blue) and two unaffected carriers (light blue). Each line represents an individual cell line.

**Fig. 4:**
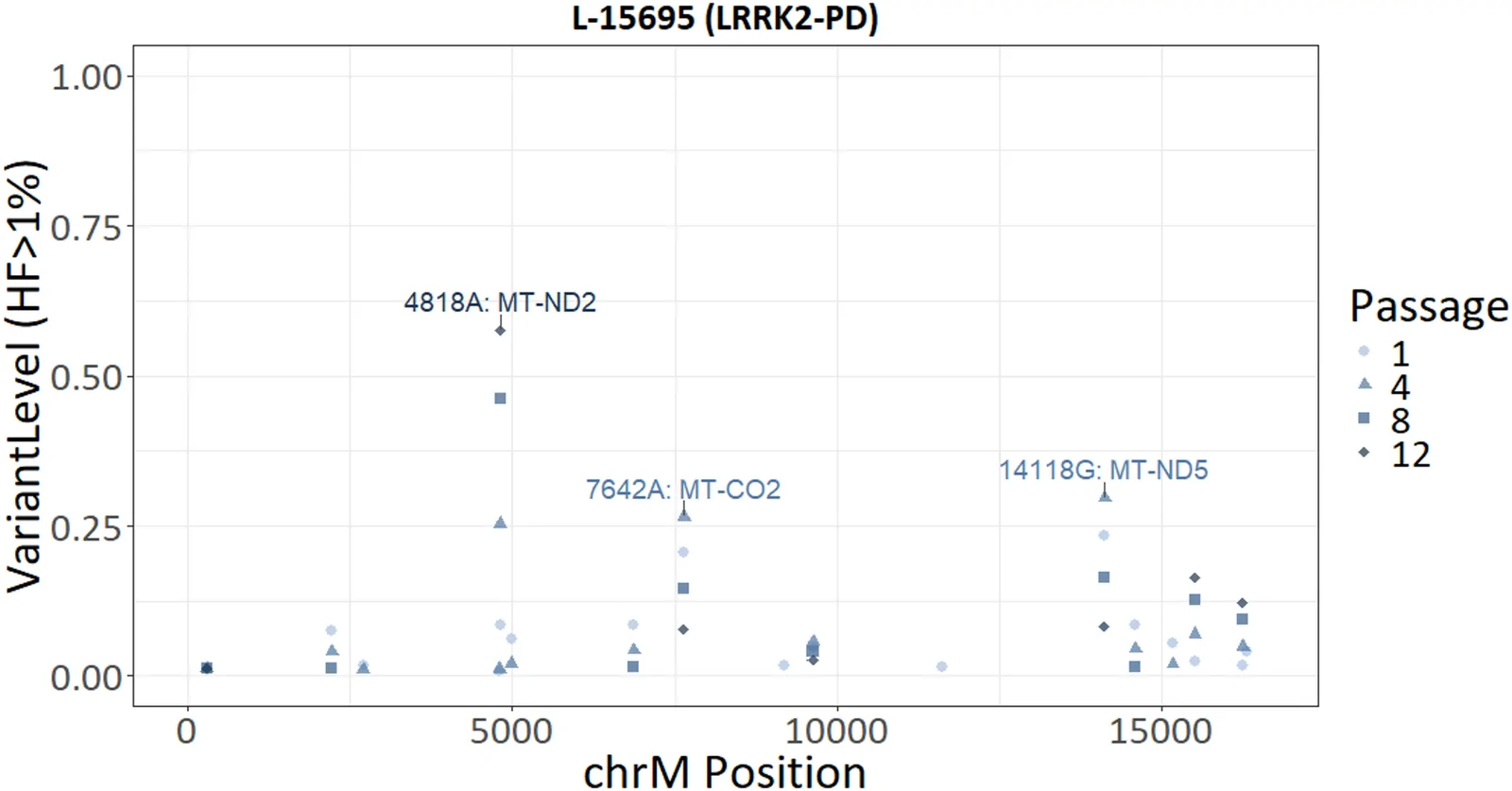
Variant-specific heteroplasmy dynamics across fibroblast passages in an LRRK2 p.Gly2019Ser carrier with Parkinson’s disease (PD). Heteroplasmy levels of mtDNA variants (> 1%) plotted across mitochondrial genome positions (chrM) for fibroblast cell line L-15,695 derived from an affected *LRRK2* variant carrier. The different symbols and colors represent heteroplasmy levels detected at fibroblast passages 1, 4, 8, and 12. Several variants exhibit distinct variant level changes across passages, including variants located in the genes *MT-ND2* (chrM:4818 A), *MT-CO2* (chrM:7642 A), and *MT-ND5* (chrM:14118G).

To apply the validated PacBio workflow to a relevant cellular model, we longitudinally assessed mitochondrial heteroplasmy in fibroblast cell lines derived from five LRRK2 p.Gly2019Ser variant carriers, three PD patients, and two unaffected carriers. Heteroplasmic variants were assessed across fibroblast passages 1 (day 0), 4 (day 28), 8 (day 56), and 12 (day 84); all cell cultures were frozen at passage 3 prior to our analysis. Based on our validation using the mixture model, we restricted the longitudinal analysis to heteroplasmic variants with heteroplasmy levels > 1%. Across the 12 passages, we observed a decrease in the number of detected heteroplasmic mtDNA variants (Fig. 3). To quantify this trend, we applied a linear mixed-effects model including passage number as the time variable, affection status, age as fixed effects, and the five individual cell lines as a random effect (Table 2). The model confirmed that over time and across passages, there were fewer heteroplasmic variants (passage 12 vs. passage 1: β=-3.2, SE = 1.3, *p* = 0.026). In exploratory analyses, mtDNA heteroplasmic variant load was higher in fibroblast lines derived from affected *LRRK2* variant carriers compared with unaffected carriers (β = 5.0, SE = 1.0, *p* = 1.0 × 10⁻⁴) and was associated with older age at sample collection (β = 1.5, SE = 0.3, *p* = 0.001). Given the small number of independent cell lines, these subject-level associations should be interpreted cautiously. As dynamic changes in mtDNA heteroplasmy were observed across fibroblast passages, variant-specific profiles were evaluated by examining mitochondrial genomic positions and heteroplasmy levels at each passage. This analysis revealed heterogeneous dynamics of specific mtDNA variants over time. While some variants gradually decreased in heteroplasmy levels across passages, others showed increasing levels, indicating variant-specific trajectories during fibroblast culture. For example, in the fibroblast line L-15,695 derived from an affected *LRRK2* carrier, we observed that the heteroplasmic variant level of chrM:7642 A (*MT-CO2*, synonymous; variant level = 20.5% to 7.7%, MITOMAP: 0.2%) and chrM:14118G (*MT-ND5*, synonymous; variant level = 23.3% to 8.3%, MITOMAP: 0.1%) decreased across passages, whereas chrM:4818 A (*MT-ND2*, p.E117K; variant level = 8.4% to 57.5%, MITOMAP: no reported frequency) showed a gradual increase (Fig. 4). Overall, heteroplasmic variant profiles were highly heterogeneous across samples and passages, with no consistent pattern observed between affected and unaffected LRRK2 p.Gly2019Ser carriers (Supplementary Fig. 4).

**Table 2:** Linear mixed-effects model assessing factors associated with the number of mtDNA heteroplasmic variants

	Estimate	Standard error	*p*-value
**Passage 4**	-1.2	1.3	0.367
**Passage 8**	-1.8	1.3	0.184
**Passage 12**	-3.2	1.3	0.026
**Affection status**: ***LRRK2*-PD**	5.0	1.0	1.0 × 10^− 4^
**Age at sample collection**	1.5	0.3	0.001

PD=Parkinson’s disease, Baseline category: Passage = 1, Affection status=*LRRK2* unaffected

R-formula: lmer (Overall.Heteroplasmies ~ Passage + Affection status + age + (1|Sample ID)); Models include fibroblast cell line as a random effect

Utilizing *Mitorsaw* and *Sniffles2* to explore structural variants like deletions, we did not detect any at an allele frequency > 1%.

## Discussion

In this study, we developed and validated a PacBio HiFi-based workflow for accurate detection of mitochondrial DNA heteroplasmy using a single full-length mtDNA amplicon. Utilizing a mitochondrial haplotype mixture model, we demonstrate very high accuracy, with 100% concordance between expected and detected variants down to the 1% variant level. Across all multiplexed samples, PacBio HiFi sequencing generated high-quality full-length reads with consistent read lengths and uniformly high coverage across the mitochondrial genome.

Sensitivity and accuracy remained high even at reduced, subsampled sequencing depth, suggesting that large numbers of samples can be multiplexed without losing heteroplasmy detection accuracy. In detail, given the sequencing depth obtained in this study, multiplexing all 230 available barcodes would still yield approximately 13,000X mtDNA coverage per sample, enabling accurate detection of mitochondrial heteroplasmy levels at 1%.

We further explored variant recovery at very low heteroplasmy levels using a mixture containing 0.1% of the minor component and a specifically lowered variant-calling threshold. Under these conditions, all expected minor-haplotype variants were recovered at approximately the expected variant level of 0.001, although a number of variants discordant with the Illumina reference were also detected at this variant-calling threshold. Two of those additional variants were located within the well-known homopolymeric D-loop region (positions 299 and 311), where alignment ambiguity is expected. The remaining discordant variants were distributed across the mitochondrial genome without evidence of systematic clustering. Read-level assessment showed that these discordant alleles were also present at low levels in the 5% mixture but remained below the higher variant-calling threshold applied to this mixture. Thus, their identification in the 0.1% analysis represents a threshold-dependent detection effect rather than variants specific to the 0.1% mixture. The discordant variants may reflect residual sequencing and alignment-related noise, in line with previous findings [11].

Furthermore, all additional discordant variants detected in the 0.1% mixtures occurred on molecules assigned to the major haplotype and were not linked to the expected minor-haplotype variants. These findings suggest that the additional variants do not arise from the minor mixture component. However, they do not allow us to distinguish between genuine low-level heteroplasmy present in the major mixture component and possible PCR- or sequence-context-dependent variant-calling errors associated with the major haplotype. Thus, while these exploratory results demonstrate recovery of the predefined minor-haplotype variants at 0.1% under the applied calling conditions, they should not be interpreted as establishing a general analytical detection limit of 0.1%.

Assessment of common variants further confirmed stable detection at homoplasmic levels (variant level ~ 1.00), indicating low technical noise. It is essential to note that the use of the Illumina dataset as a gold-standard reference may overestimate the number of discordant variants at very low variant levels, as orthogonal validation (e.g., sequencing of unmixed samples) was not performed in this study.

Beyond accurate detection of individual heteroplasmic/minor variants, PacBio HiFi sequencing enabled direct reconstruction of complete mitochondrial haplotypes at the single-molecule level. Informative haplotype-specific variants consistently co-occurred on individual full-length reads across all predefined haplotype mixtures, demonstrating that variants originating from the same mitochondrial genome can be accurately phased. This illustrates an important advantage of long-read sequencing over short-read approaches, which generally might not be suitable to resolve full-length mitochondrial haplotypes directly.

To our knowledge, we are the first to demonstrate the applicability of PacBio HiFi sequencing for highly accurate detection of mitochondrial heteroplasmy by integrating real sequencing data with a haplotype mixture model. Previous studies have explored the accuracy of mtDNA heteroplasmic variant calling using long-read sequencing technologies, primarily ONT. Reliable detection of relatively high heteroplasmy levels > 12% [27] or > 10% [28] were reported, but partially with lower sequencing depth [27].

In our previous work, we evaluated the accuracy of mtDNA heteroplasmy variant calling using deep, targeted mtDNA ONT sequencing across two overlapping PCR amplicons. In contrast, we demonstrated reliable mtDNA heteroplasmy variant calling > 5% variant levels [11]. However, the overall accuracy for very low-frequency variants below 5% remained limited with ONT sequencing using the flow cell and basecalling models available at that time (R9.4.1 chemistry and Guppy v5.0.11 basecalling). This work does not compare the most recent chemistry of ONT to PacBio. Here, we demonstrate that PacBio HiFi sequencing enables highly accurate detection of mitochondrial heteroplasmy between 1% and 5% using the same benchmarking framework previously applied to ONT sequencing. While recent improvements in ONT chemistry and basecalling are expected to enhance low-frequency variant detection, a direct comparison between current ONT and PacBio HiFi technologies was beyond the scope of the present study.

The precise assessment of low-frequency variants is important for various research fields, including cancer research and the investigation of mosaic somatic variants and of course mitochondrial heteroplasmy [2, 5, 29, 30]. More broadly, genome-wide benchmarking studies of somatic variant detection have reported lower F_1_ scores and recall-rates/sensitivity with lower whole-genome sequencing depths (~ 40X-60X) [31–33]. This highlights the advantage of the targeted high-depth sequencing approaches for detecting rare variants in the mtDNA.

Some limitations of the presented workflow are important to note. The use of long-range PCR introduces the risk of primer dropout if polymorphisms occur within the primer binding sites, which may lead to complete amplification failure. Most prominently Haplogroup: D1, H1n, M24b, U3, U7, and T2a, which all have known polymorphisms at chrM:2090–2150. As primer sequences were not removed prior to variant calling, variants within the primer-binding regions cannot be reliably assessed and are therefore excluded from interpretation. However, none of the predefined major, minor, or common variants used for benchmarking were located within the primer-binding regions. Importantly, as the predefined mixtures were generated after separate long-range PCR amplification, the benchmarking experiment does not assess PCR sampling effects, amplification bias, polymerase errors, or allele-specific amplification at very low heteroplasmy levels. PCR amplification can also introduce low variant-level polymerase errors that might have been missed in the present benchmarking. In addition, PCR-based enrichment removes native DNA methylation, and thus, analysis of mitochondrial epigenetic architecture is not possible with our workflow. Furthermore, our validation focused on single-nucleotide variant detection. The performance of this workflow for structural variant detection was not systematically evaluated. Although the use of a single full-length mtDNA amplicon may, in principle, facilitate structural variant detection compared with multi-amplicon approaches, long-range PCR itself introduces inherent limitations. For example, heteroplasmy levels may be influenced by PCR amplification bias, and structural variants disrupting the primer-binding sites may not be amplified and therefore remain undetected, as previously described [34]. While we explored the presence of structural variants, such as deletions, none were detected in the analyzed samples. This likely reflects the absence of structural variants above the applied detection threshold (> 1%) rather than a limitation of the approach. Consistent with this, the read length distribution of the unfiltered sequencing data did not show an enrichment of shorter reads, as would be expected in the presence of deletion-containing molecules. In addition, the long-range PCR strategy together with read-length and alignment-length filtering is expected to substantially reduce interference from fragmented nuclear mitochondrial insertions (NUMTs), which can be a more prominent concern in whole-genome and short-read sequencing approaches.

Finally, as a proof-of-principle application of the validated workflow, we investigated longitudinal mtDNA heteroplasmy in fibroblasts derived from LRRK2 p.Gly2019Ser variant carriers. Based on the results from the mixture validation, we focused on heteroplasmic variants with variant levels > 1%.

Overall, we observed a decrease in the mitochondrial heteroplasmic variant load across all cell lines over time. Indeed, evaluating the individual positions and levels of detected heteroplasmic variants showed that certain variants exhibit a general decrease in their levels over time. There were even variants that were detected in passages 1 to 8 but no longer in passage 12 (Fig. 4). However, other variants exhibit a gradual increase in variant levels across passages (e.g. chrM:4818 A in *MT-ND2*, Fig. 4). Taken together, this may suggest that selective pressures or clonal expansion processes are shaping mitochondrial heteroplasmy during fibroblast passaging. A recent study investigated mtDNA heteroplasmy dynamics with a single-cell approach at different cell culture conditions. It was demonstrated that heteroplasmy levels in dividing cells are primarily shaped by selection rather than random drift [35]. Furthermore, deleterious mtDNA variants can be selectively removed or not, depending on improvements in cell fitness under different cell culture conditions [35]. Similarly, a study investigating mtDNA heteroplasmy during iPSC reprogramming and passaging reported no evidence for the emergence of novel heteroplasmic variants but observed that selection during passaging contributes to shaping heteroplasmy [36]. It is important to note that we did not detect a shared pattern of dynamically changing variants across all analyzed samples, indicating substantial inter-individual variability in heteroplasmy trajectories.

We investigated heteroplasmy over time in fibroblasts derived from three *LRRK2* variant carriers affected with PD and two unaffected carriers, and the heteroplasmic variant load was higher in fibroblasts from affected than from unaffected carriers. Mitochondrial dysfunction is a key player in PD pathogenesis, including impairment of mtDNA integrity [37]. Thus, despite the limited sample size, our observations are consistent with the growing body of evidence linking increased mtDNA heteroplasmy or damage to *LRRK2*-related PD, as highlighted by previous studies [13–15]. Notably, somatic major arc deletions have been reported to be increased in fibroblast cultures derived from *LRRK2*-PD patients and to discriminate affected from unaffected variant carriers [15]. To our knowledge, this exploratory proof-of-principle study is among the first to longitudinally assess mtDNA heteroplasmy dynamics across fibroblast passages using high-accuracy long-read sequencing in the context of *LRRK2*-related PD, providing new insights into variant-specific trajectories and the role of selection in shaping mitochondrial variation over time.

Future studies in larger, independent cohorts are required to comprehensively characterize mitochondrial heteroplasmy in *LRRK2*-related PD and other PD subtypes using long-read sequencing approaches.

## Conclusion

PacBio HiFi sequencing combined with a single-amplicon strategy facilitates accurate full-length mtDNA heteroplasmy detection and longitudinal analysis. Our benchmarking demonstrates highly accurate detection of heteroplasmic variants down to 1%, while detection at 0.1% retained high sensitivity but was affected by variants discordant with the reference set. Application of this workflow to *LRRK2* p.Gly2019Ser variant carrier fibroblast models revealed dynamic, variant-specific changes in heteroplasmy over time, and an association between higher heteroplasmic variant load and *LRRK2*-PD. Together, this approach provides a powerful tool to study mitochondrial heteroplasmy and its role in aging and disease.

## Supplementary information

Below is the link to the electronic supplementary material.

Supplementary Material 1

## Data Availability

The unfiltered FASTQ sequencing files supporting the conclusions and generated for this article are available in the Sequence Read Archive (SRA https://www.ncbi.nlm.nih.gov/sra) under the accession number: PRJNA1533167 (SAMN63302188-SAMN63302211).

## Abbreviations

AF: Allele Frequency
BAM: Binary Alignment Map
bp: Base Pairs
chrM: Mitochondrial Chromosome
DNA: Deoxyribonucleic Acid
F1 score: Harmonic Mean of Precision and Sensitivity
HiFi: High-Fidelity
iPSC: Induced Pluripotent Stem Cell
kb: Kilobase
LRRK2: Leucine-Rich Repeat Kinase 2
mtDNA: Mitochondrial DNA
ONT: Oxford Nanopore Technology
PCR: Polymerase Chain Reaction
PD: Parkinson's Disease
Q-score: Phred Quality Score
rCRS: Revised Cambridge Reference Sequence
SE: Standard Error
SNV: Single Nucleotide Variant
SV: Structural Variant
VAF: Variant Allele Frequency
X: Sequencing Coverage Depth
