# Supplementary material for: Long-read sequencing enables high-accuracy mitochondrial heteroplasmy detection in Parkinson’s disease
Source: BMC Genomics. 2026 Oct 8;27:831. doi: 10.1186/s12864-026-13426-y (PMC13651137; doi:10.1186/s12864-026-13426-y)
Supplement: Supplementary file 1 [file 12864_2026_13426_MOESM1_ESM.docx]

**Supplementary material:**

**Supplementary Table 1. Overview of samples used for the mixture model and in the fibroblast assessment.**

| **Mixture model** | | | | |
| --- | --- | --- | --- | --- |
| **ID** | **Age at sample collection** | **Ancestry & Country** | **Mitochondrial haplogroup** | |
| **B-28** | 37 | European / Italy | D4e1’3 | |
| **L-2804** | 75 | European / Germany | J1c2 | |
| **Fibroblast cell lines derived from LRRK2 p.Gly2019Ser variant carriers** | | | | |
| **ID** | **Age at sample collection** | **Ancestry & Country** | **Affection status** | **Mitochondrial haplogroup** |
| **L-15695** | 54 | European / Germany | Parkinson’s disease | H5 |
| **L-20393** | 53 | European / Germany | Parkinson’s disease | H5a1b |
| **L-15935** | 51 | European / Germany | Parkinson’s disease | T1a1k |
| **L-3086** | 55 | European / Germany | Unaffected | J1c2p |
| **L-3085** | 52 | European / Germany | Unaffected | J1c2p |

**Supplementary Table 2. Mitochondrial DNA Long-range PCR mix for one sample and cycling conditions for amplifying the single mitochondrial amplicon.**

| **PCR master-mix** | |
| --- | --- |
| **Reagent** | **1x [µl]** |
| SuperFi II OCR MM | 12.5 |
| 10µM Primer F | 1.25 |
| 10µM Primer R | 1.25 |
| DNA (50ng) | X |
| Nuclease-free water | 10.0-X |
| ∑ | 25.0 |
| **Cycler conditions** | |
| **Temperature and duration** | **Process and cycles** |
| 94°C for 1:00 | Initial denaturation |
| 98°C for 0:10 | 30 cycles |
| 68°C for 16:00 |  |
| 72°C for 10:00 | Final extension |
| 4°C forever | ---- |

**Supplementary Table 3. Preparation of the mixtures of the PCR products at predefined 5%, 2%, 1% and 0.1% ratios.**

|  | | **Major component: D4e1’3** | **Minor component: J1c2** |
| --- | --- | --- | --- |
| **Volume of PCR product [µl]** | **0.1%** | 49.5 | 0.5* |
|  | **1%** | 49.5 | 0.5 |
|  | **2%** | 49.0 | 1.0 |
|  | **5%** | 47.5 | 2.5 |

*1:10 dilution of the minor component

**Supplementary Table 4. Overview of sequencing quality parameters across all multiplexed samples.**

| **Sample** | **Median Q score** | **Sequencing depth**  **[X]** | **N50**  **[bp]** |
| --- | --- | --- | --- |
| **Mix 5%** | 30.90 | 64035 | 16565.00 |
| **Mix 2%** | 30.80 | 66009 | 16565.00 |
| **Mix 1%** | 30.80 | 75179 | 16565.00 |
| **Mix 0.1%** | 30.90 | 70154 | 16565.00 |
| **Fibros_L-3086_P1** | 30.90 | 65663 | 16564.00 |
| **Fibros_L-15695_P1** | 30.80 | 73651 | 16566.00 |
| **Fibros_L-15935_P1** | 30.90 | 91115 | 16565.00 |
| **Fibros_L-20393_P1** | 30.90 | 83801 | 16562.00 |
| **Fibros_L-3086_P4** | 30.90 | 83099 | 16564.00 |
| **Fibros_L-15695_P4** | 30.80 | 81542 | 16566.00 |
| **Fibros_L-15935_P4** | 30.90 | 88923 | 16565.00 |
| **Fibros_L-20393_P4** | 30.90 | 92396 | 16562.00 |
| **Fibros_L-3086_P8** | 30.90 | 81847 | 16564.00 |
| **Fibros_L-15695_P8** | 30.90 | 87691 | 16566.00 |
| **Fibros_L-15935_P8** | 30.80 | 95769 | 16565.00 |
| **Fibros_L-20393_P8** | 30.90 | 79959 | 16562.00 |
| **Fibros_L-3086_P12** | 31.00 | 86260 | 16564.00 |
| **Fibros_L-15695_P12** | 30.90 | 72746 | 16566.00 |
| **Fibros_L-15935_P12** | 30.90 | 78948 | 16565.00 |
| **Fibros_L-20393_P12** | 30.90 | 86547 | 16562.00 |
| **Fibros_L-3085_P1** | 31.00 | 107279 | 16564.00 |
| **Fibros_L-3085_P4** | 31.00 | 97562 | 16564.00 |
| **Fibros_L-3085_P8** | 31.00 | 99115 | 16564.00 |
| **Fibros_L-3085_P12** | 30.90 | 87028 | 16564.00 |

**Supplementary Table 5. Performance of mtDNA heteroplasmy detection across sequencing depths in predefined haplotype mixtures following subsampling of PacBio HiFi sequencing data, with N=5 replicates for each subsampling step.**

| **5% Mixture** | | | | | |
| --- | --- | --- | --- | --- | --- |
| **Target coverage** | **Mean coverage**  **[X]** ±**SD** | **Mean detected minor variants* (range)** | **Mean additional variant calls (range)** | **Mean sensitivity** ± **SD** | **Mean F_1_ score** ±**SD** |
| 300 | 300 ± 20 | 18 (18-18) | 0 (0-0) | 1.00 ± 0.00 | 1.00 ± 0.00 |
| 500 | 500 ± 23 | 18 (18-18) | 0 (0-0) | 1.00 ± 0.00 | 1.00 ± 0.00 |
| 800 | 792 ±11 | 18 (18-18) | 0 (0-0) | 1.00 ± 0.00 | 1.00 ± 0.00 |
| 1000 | 999 ± 16 | 18 (18-18) | 0 (0-0) | 1.00 ± 0.00 | 1.00 ± 0.00 |
| 1500 | 1499 ± 18 | 18 (18-18) | 0 (0-0) | 1.00 ± 0.00 | 1.00 ± 0.00 |
| 2000 | 2008 ± 31 | 18 (18-18) | 0 (0-0) | 1.00 ± 0.00 | 1.00 ± 0.00 |
| 3000 | 3000 ± 57 | 18 (18-18) | 0 (0-0) | 1.00 ± 0.00 | 1.00 ± 0.00 |
| 5000 | 4997 ± 102 | 18 (18-18) | 0 (0-0) | 1.00 ± 0.00 | 1.00 ± 0.00 |
| 10000 | 10032 ± 134 | 18 (18-18) | 0 (0-0) | 1.00 ± 0.00 | 1.00 ± 0.00 |
| 20000 | 20001 ± 165 | 18 (18-18) | 0 (0-0) | 1.00 ± 0.00 | 1.00 ± 0.00 |
| 40000 | 39979 ± 74 | 18 (18-18) | 0 (0-0) | 1.00 ± 0.00 | 1.00 ± 0.00 |
| Full data set** | 64039 | 18 | 0 | 1.00 | 1.00 |
| **2% Mixture** | | | | | |
|  | | | | | |
| 300 | 298 ± 9 | 6.2 (0-16) | 0.20 (0-1) | 0.77 ± 0.16 | 0.86 ± 0.10 |
| 500 | 501 ± 10 | 14.0 (0-18) | 0.40 (0-1) | 0.92 ± 0.15 | 0.95 ± 0.09 |
| 800 | 811 ± 17 | 18 (18-18) | 0.60 (0-1) | 1.00 ± 0.00 | 0.99 ± 0.01 |
| 1000 | 999 ± 21 | 18 (18-18) | 0.60 (0-1) | 1.00 ± 0.00 | 0.99 ± 0.01 |
| 1500 | 1506 ± 27 | 18 (18-18) | 0.60 (0-1) | 1.00 ± 0.00 | 0.99 ± 0.01 |
| 2000 | 2004 ± 51 | 18 (18-18) | 0.40 (0-1) | 1.00 ± 0.00 | 1.00 ± 0.01 |
| 3000 | 2996 ± 57 | 18 (18-18) | 0.60 (0-1) | 1.00 ± 0.00 | 0.99 ± 0.01 |
| 5000 | 4971 ± 85 | 18 (18-18) | 0.20 (0-1) | 1.00 ± 0.00 | 1.00 ± 0.00 |
| 10000 | 9953 ± 56 | 18 (18-18) | 0.40 (0-1) | 1.00 ± 0.00 | 1.00 ± 0.01 |
| 20000 | 19955 ± 107 | 18 (18-18) | 0.40 (0-1) | 1.00 ± 0.00 | 1.00 ± 0.01 |
| 40000 | 39921 ± 126 | 18 (18-18) | 0.20 (0-1) | 1.00 ± 0.00 | 1.00 ± 0.00 |
| Full data set** | 66013 | 18 | 0 | 1.00 | 1.00 |
| **1% Mixture** | | | | | |
| 300 | 308 ± 19 | 3.4 (0-16) | 0.20 (0-1) | 0.72 ± 0.14 | 0.83 ± 0.08 |
| 500 | 511 ± 30 | 12.0 (1-18) | 0.40 (0-1) | 0.88 ± 0.15 | 0.93 ± 0.09 |
| 800 | 822 ± 40 | 14.4 (1-18) | 1.00 (0-2) | 0.93 ± 0.15 | 0.95 ± 0.09 |
| 1000 | 1030 ± 43 | 11.6 (1-18) | 1.20 (0-2) | 0.88 ± 0.17 | 0.92 ± 0.11 |
| 1500 | 1525 ± 62 | 18 (18-18) | 1.20 (0-2) | 1.00 ± 0.00 | 0.99 ± 0.01 |
| 2000 | 2028 ± 60 | 18 (18-18) | 1.80 (0-3) | 1.00 ± 0.00 | 0.98 ± 0.01 |
| 3000 | 3032 ± 71 | 18 (18-18) | 1.60 (1-3) | 1.00 ± 0.00 | 0.99 ± 0.01 |
| 5000 | 4978 ± 90 | 17.8 (17-18) | 2.20 (1-3) | 1.00 ± 0.01 | 0.98 ± 0.01 |
| 10000 | 9981 ± 79 | 18 (18-18) | 1.60 (1-2) | 1.00 ± 0.00 | 0.99 ± 0.01 |
| 20000 | 19988 ± 92 | 18 (18-18) | 2.20 (2-3) | 1.00 ± 0.00 | 0.98 ± 0.00 |
| 40000 | 40007 ± 114 | 18 (18-18) | 1.60 (1-3) | 1.00 ± 0.00 | 0.99 ± 0.01 |
| Full data set** | 75183 | 18 | 0 | 1.00 | 1.00 |
| **0.1% Mixture** | | | | | |
| 300 | 292 ± 22 | 0.00 (0-0) | 0.20 (0-1) | 0.65 ± 0.00 | 0.79 ± 0.004 |
| 500 | 498 ± 26 | 0.00 (0-0) | 0.20 (0-1) | 0.65 ± 0.00 | 0.79 ± 0.004 |
| 800 | 796 ± 40 | 0.00 (0-0) | 0.20 (0-1) | 0.65 ± 0.00 | 0.79 ± 0.004 |
| 1000 | 1006 ± 47 | 0.00 (0-0) | 0.20 (0-1) | 0.65 ± 0.00 | 0.79 ± 0.004 |
| 1500 | 1493 ± 49 | 0.00 (0-0) | 0.80 (0-2) | 0.65 ± 0.00 | 0.79 ± 0.01 |
| 2000 | 1975 ± 51 | 0.20 (0-1) | 1.60 (1-2) | 0.66 ± 0.01 | 0.78 ± 0.01 |
| 3000 | 2967 ± 43 | 0.60 (0-2) | 4.00 (2-5) | 0.67 ± 0.02 | 0.76 ± 0.01 |
| 5000 | 4971 ± 60 | 0.20 (0-1) | 6.80 (6-9) | 0.66 ± 0.01 | 0.74 ± 0.01 |
| 10000 | 9991 ± 54 | 9.80 (0-16) | 10.80 (8-14) | 0.84 ± 0.13 | 0.82 ± 0.09 |
| 20000 | 19992 ± 21 | 14.00 (1-18) | 9.80 (9-10) | 0.92 ± 0.14 | 0.87 ± 0.08 |
| 40000 | 40033 ± 75 | 12.40 (6-18) | 9.00 (8-11) | 0.89 ± 0.11 | 0.86 ± 0.07 |
| Full data set** | 70158 | 18 | 10 | 1.00 | 0.91 |

*N=18 expected minor variants; **Full data set uses all sequencing data and no sub-sampling was done, consequently this is an observation of N=1 and the calculation of mean/SD is not applicable; F_1_ score = 2 × (precision × sensitivity) ÷ (precision + sensitivity); Sensitivity = (Number of true positive calls) ÷ (Number of true positive calls + Number of false negative calls); Precision = (Number of true positive calls) ÷ (Number of true positive calls + Number of false positive calls)

| **Position** | **Region/ Locus** | **Ref** | **Alt** | **Genomic context +/- 10 bp** | **Variant MITOMAP frequency** | **Variant level** | **Variant level (*Mitorsaw)*** |
| --- | --- | --- | --- | --- | --- | --- | --- |
| **299** | D-loop | C | A | AAATTTCCAC**-C-**AAACCCCCCC | 0.010% | 0.001 | 0.001 |
| **311** | D-loop | C | T | AACCCCCCCT**-C-**CCCCGCTTCT | 0.037% | 0.001 | - |
| **1419** | RNR1 | G | A | AGGGTCGAAG**-G-**TGGATTTAGC | 0.000% | 0.003 | 0.006 |
| **8078** | MT-CO2 | G | A | CTCATGAGCT**-G-**TCCCCACATT | 0.045% | 0.002 | - |
| **10063** | MT-ND3 | A | G | AGAGTAATAA**-A-**CTTCGCCTTA | No variant ref frequency available on UCSC | 0.001 | - |
| **10196** | MT-ND3 | C | G | CTATATCCCC**-C-**GCCCGCGTCC | No variant ref frequency available on UCSC | 0.001 | - |
| **10197** | MT-ND3 | G | C | TATATCCCCC**-G-**CCCGCGTCCC | 0.000 | 0.001 | - |
| **12467** | MT-ND5 | T | C | GCATCCACCT**-T-**TATTATCAGT | 0.002% | 0.005 | - |
| **15249** | MT-CYB | A | G | TGAGGAGGCT**-A-**CTCAGTAGAC | 0.002% | 0.001 | - |
| **15302** | MT-CYB | C | A | CTTCATCTTG**-C-**CCTTCATTAT | No variant ref frequency available on UCSC | 0.002 | - |

**Supplementary Table 6: Summary of mitochondrial variants discordant with the Illumina reference identified from the 0.1% mixture and regional relevance. All coordinates correspond to GRCh38/hg38 mitochondrial reference sequence in UCSC. MITOMAP frequencies are reported from GenBank full-length sequences.**

**Supplementary Table 7: Summary of common variants (i.e., shared variants between major and minor mixture components). Example data of the 5% mixture are displayed.**

| **Full data set (at 64,039X sequencing depth)** | | | |
| --- | --- | --- | --- |
| **Position** | **Reference** | **Variant** | **Variant level** |
| 73 | A | G | 1 |
| 263 | A | G | 1 |
| 489 | T | C | 1 |
| 750 | A | G | 1 |
| 1438 | A | G | 1 |
| 2706 | A | G | 1 |
| 3010 | G | A | 1 |
| 4769 | A | G | 1 |
| 7028 | C | T | 1 |
| 8860 | A | G | 1 |
| 10398 | A | G | 1 |
| 11719 | G | A | 1 |
| 14766 | C | T | 1 |
| 15326 | A | G | 1 |
| **Subsampled data set (0.005 fraction of sequenced data at 311X sequencing depth)** | | | |
| 73 | A | G | 1 |
| 263 | A | G | 1 |
| 489 | T | C | 1 |
| 750 | A | G | 1 |
| 1438 | A | G | 1 |
| 2706 | A | G | 0.997 |
| 3010 | G | A | 1 |
| 4769 | A | G | 1 |
| 7028 | C | T | 0.997 |
| 8860 | A | G | 1 |
| 10398 | A | G | 1 |
| 11719 | G | A | 1 |
| 14766 | C | T | 1 |
| 15326 | A | G | 1 |

**Supplementary Table 8. Read-level haplotype classification across predefined mitochondrial DNA haplotype mixtures.**

| **Expected minor haplotype fraction** | **Total analyzed reads** | **Minor-haplotype reads (%)** | **Observed minor-read percentage** |
| --- | --- | --- | --- |
| **5%** | 64,121 | 3,677 | 5.7% |
| **2%** | 66,098 | 1,459 | 2.2% |
| **1%** | 75,288 | 807 | 1.1% |
| **0.1%** | 70,255 | 60 | 0.1% |

**Supplementary Figure 1. Exemplary distribution of read lengths before and after filtering.** Histograms show the distribution of sequencing read lengths for unfiltered (bottom) and filtered (top) datasets. Read lengths are measured in base pairs (bp)

**Supplementary Figure 2. Sequencing depth across the mitochondrial genome, resulting in the single amplicon long-range PCR and PaBio sequencing.** Coverage across mitochondrial genome positions (chrM) for the 5% haplotype mixture using the full dataset (~64,000× depth, **(A)**) and after subsampling to ~300× depth **(B)**.

**Supplementary Figure 3. Read-level haplotype assignment of full-length PacBio HiFi sequencing reads from the 1% mitochondrial DNA haplotype mixture.** Representative full-length PacBio HiFi reads were classified according to informative haplotype-specific variants. Ten reads assigned to the major haplotype and ten reads assigned to the minor haplotype are shown. Columns represent informative mitochondrial DNA positions, and rows represent individual sequencing molecules. The consistent co-occurrence of haplotype-specific variants across complete sequencing molecules demonstrates reconstruction of full-length mitochondrial haplotypes.

**Supplementary Figure 4.** **Heteroplasmy dynamics across fibroblast passages.** Heteroplasmic variants are plotted across mitochondrial genome positions for fibroblast cell lines derived from LRRK2 p.G2019S carriers. Panels represent individual cell lines, and symbols/colors indicate passages 1, 4, 8, and 12.
